# A Review of the Mechanisms of Blood-Brain Barrier Permeability by Tissue-Type Plasminogen Activator Treatment for Cerebral Ischemia

**DOI:** 10.3389/fncel.2016.00002

**Published:** 2016-01-25

**Authors:** Yasuhiro Suzuki, Nobuo Nagai, Kazuo Umemura

**Affiliations:** ^1^Department of Pharmacology, Hamamatsu University School of MedicineHamamatsu, Japan; ^2^School of Pharmaceutical Sciences, Ohu UniversityKoriyama, Japan; ^3^Faculty of Bioscience, Department of Animal Bioscience, Nagahama Institute of Bio-Science and TechnologyNagahama, Japan

**Keywords:** brain ischemia, blood-brain barrier permeability, endothelial endocytosis, intracranial bleeding, tissue-type plasminogen activator, vascular endothelial growth factor

## Abstract

Cerebrovascular homeostasis is maintained by the blood-brain barrier (BBB), which forms a mechanical and functional barrier between systemic circulation and the central nervous system (CNS). In patients with ischemic stroke, the recombinant tissue-type plasminogen activator (rt-PA) is used to accelerate recanalization of the occluded vessels. However, rt-PA is associated with a risk of increasing intracranial bleeding (ICB). This effect is thought to be caused by the increase in cerebrovascular permeability though various factors such as ischemic reperfusion injury and the activation of matrix metalloproteinases (MMPs), but the detailed mechanisms are unknown. It was recently found that rt-PA treatment enhances BBB permeability not by disrupting the BBB, but by activating the vascular endothelial growth factor (VEGF) system. The VEGF regulates both the dissociation of endothelial cell (EC) junctions and endothelial endocytosis, and causes a subsequent increase in vessel permeability through the VEGF receptor-2 (VEGFR-2) activation in ECs. Here, we review the possibility that rt-PA increases the penetration of toxic molecules derived from the bloodstream including rt-PA itself, without disrupting the BBB, and contributes to these detrimental processes in the cerebral parenchyma.

## Introduction

The recombinant tissue-type plasminogen activator (rt-PA), a serine proteinase, is a thrombolytic agent that degrades fibrin clots through the activation of plasminogen to plasmin (Lijnen and Collen, 1987). Although rt-PA given within 3 h from the onset of ischemic stroke improves patients’ clinical outcome, it induces a 10-fold increase of symptomatic intracranial hemorrhage (The National Institute of Neurological Disorders and Stroke rt-PA Stroke Study Group, 1995). Furthermore, rt-PA treatment delayed beyond 3 h is associated with an increased risk of hemorrhagic transformation with enhanced brain injury (Clark et al., 1999). Subsequently, the European Cooperative Acute Stroke Study showed that rt-PA administered between 3 and 4.5 h after the onset of symptoms significantly improved the clinical outcomes of patients with acute ischemic stroke, but it increased the risk of symptomatic intracranial hemorrhage. It was confirmed that delayed treatment beyond 4.5 h was not associated with a statistically significant benefit (Hacke et al., 2008). A number of clinical studies using magnetic resonance imaging provided evidence that rt-PA treatment is associated with blood-brain barrier (BBB) breakdown (Kastrup et al., 2008; Kassner et al., 2009), which also correlates with an increased risk of hemorrhagic transformation during thrombolysis in ischemic stroke (Kassner et al., 2009). These findings strongly suggest a causal relationship between rt-PA and BBB breakdown in the ischemic human brain.

Although the deleterious effect of rt-PA after ischemic stroke has been widely accepted, it remains unclear whether many blood-derived rt-PAs penetrate the brain and contribute to these detrimental processes in the cerebral parenchyma. Intravenously administered rt-PA has been shown to cross brain endothelial cells (ECs) via two ways: (1) by binding to the surface; and (2) transcytosis without compromising the BBB integrity (Benchenane et al., 2005; López-Atalaya et al., 2007). rt-PA was also found to enter the parenchyma under pathological conditions where it further affects BBB breakdown (Su et al., 2008). Some hypotheses have been proposed to explain how rt-PA within the parenchyma exacerbates intracranial bleeding (ICB) after ischemic stroke. Extracellular rt-PA can mediate some of its actions either through plasmin; the degradation of laminin, one of the extracellular matrix (ECM) proteins (Chen and Strickland, 1997); the activation of microglia (Rogove and Tsirka, 1998); or the excessive induction of vascular remodeling and angiogenesis via overactivation of metalloproteinases (MMPs; Suzuki et al., 2007; Yamashita et al., 2009; Won et al., 2014) and the vascular endothelial growth factor (VEGF; Kanazawa et al., 2011; Suzuki et al., 2015). There is also evidence that rt-PA may have a direct toxic effect on the ischemic brain (Wang et al., 1998; Nagai et al., 1999), possibly through activation of the N-methyl-D-aspartate receptor (NMDAR; Nicole et al., 2001). This may be of particular importance given that rt-PA diffused within the cerebral parenchyma after ischemia can have detrimental effects including enhancing the neurotoxic processes (Kaur et al., 2004).

In the present review, we firstly described BBB breakdown by rt-PA, and then discussed the role of the enhancement of BBB permeability without compromising BBB integrity on BBB breakdown. Especially, we focus on the involvement of VEGF in ECs as the first step in BBB breakdown by the deleterious effect of rt-PA after ischemic stroke. In addition, it is described the possibility that an inhibition the enhancement of BBB permeability without compromising BBB integrity may extend the therapeutic time widow by rt-PA for ischemic stroke.

## BBB and Endothelial Tight Junctions

The BBB is formed by endothelial tight junctions (TJs) together with pericytes, perivascular astrocytes, and basement membrane in the vasculature. Furthermore, as cerebrovascular function is regulated by the neuronal environment, the BBB and neurons form a functional unit called the neurovascular unit. TJs are constituted by multiple protein components that involve transmembrane proteins (e.g., occludin, claudins, and junction-associated molecules) linked to the actin cytoskeleton via cytoplasmic zonula occludens (ZO) proteins. Transmembrane proteins, occludin and claudins are critical for paracellular function at the BBB (Hawkins and Davis, 2005). Claudins are small transmembrane proteins (20–24 kDa) that span the membrane four times; claudin-1, -3, and -5 are expressed in ECs of the BBB. Occludin is a 60–65 kDa phosphoprotein highly expressed in cerebral endothelia, but it is sparsely distributed in peripheral endothelia (Hirase et al., 1997). The overexpression of claudins can induce the formation of TJs, but the expression of occludin does not lead to the formation of TJs. Thus, it is likely that claudins form the primary seal of TJs, and occludin acts as an additional support structure. Claudin and occludin are anchored to the actin cytoskeleton via ZO-1 (Hawkins and Davis, 2005).

### rt-PA and Reperfusion Injury

In patients with ischemic stroke, rt-PA increases the risk of ICB via BBB breakdown through a number of mechanisms. One of these mechanisms is thought to be by reperfusion after the degradation of occlusive blood clots by rt-PA. Brain parenchymal damage occurs because of a complex series of events in the setting of ischemia followed by reperfusion injury. These events start due to an interruption in blood flow to the affected tissue followed by the depletion of cellular energy resources and glycolysis at an anerobic substrate level, with subsequent lactic acidosis, failure of the sodium potassium pump, the release of glutamate, cytotoxic edema, and free radical formation (Nour et al., 2013). The activation of both innate and adaptive immune responses also creates free radicals. This excessive generation of free radicals overwhelms the system, which then becomes inefficient in scavenging these molecules, leading to BBB breakdown. Furthermore, ICB associated with ischemic infarction is recognized due to ischemia followed by reperfusion, and both the rate of ICB and stroke outcome can be increased by the duration of reperfusion from the onset of vessel occlusion due to ischemic stroke (Jickling et al., 2014). Additional injury is extensively shown in the brain and in other tissues, which is mediated by reactive radical oxide species (ROS). ROS contribute to BBB disruption by several mechanisms: oxidative damage to cellular molecules (i.e., proteins, lipids, and DNA); the activation of MMPs and subsequent degradation of basement membrane; cytoskeletal reorganization of ECs; the modulation of TJ proteins and upregulation of inflammatory mediators; and subsequent additional and extensive reperfusion injury (Kahles and Brandes, 2012).

### rt-PA Promotes ICB Through Mechanisms Beyond its Role in Thrombolysis and Reperfusion

rt-PA increases BBB permeability via degradations of basement membrane and TJ proteins. These degradations are associated with plasmin activation, low density lipoprotein receptor associated protein-1 (LRP-1) stimulation, and MMPs induction (Yamashita et al., 2009; Won et al., 2014). As a result, rt-PA exacerbates ischemic brain damage and ICB by increasing BBB permeability.

Plasmin, which is activated by rt-PA, can directly degrade fibrin clots and basement membrane components such as collagen IV (Mackay et al., 1990; Lukic-Panin et al., 2010), laminin (Chen and Strickland, 1997) and fibronectin (Marchina and Barlati, 1996), or via the activation of MMPs (Lijnen, 2001), which possess similar basement membrane dismantling capabilities and damages the TJs (Jin et al., 2010).

LRP is one of the major binding sites of rt-PA (Bu et al., 1992) on the cell surface. LRP, a member of the lipoprotein receptor family, is a scavenger receptor that binds a variety of biological ligands associated with the ECM and is thought to be primarily involved in lipoprotein metabolism (Herz et al., 1988), and in the clearance of protease-inhibitor complexes in the adult brain (Bu et al., 1992). Furthermore, the increase in BBB permeability by rt-PA occurs via the activation of LRP (Yepes et al., 2003; Benchenane et al., 2005; Su et al., 2008; Suzuki et al., 2009; Niego and Medcalf, 2014). LRP is selectively upregulated in ECs under ischemic stress, and LRP activation by binding to rt-PA stimulates signal pathways such as the nuclear factor κB pathway (Suzuki et al., 2009).

MMPs, a family of zinc endopeptidases, contribute to tissue remodeling through the degradation of ECM proteins. Although clarifying the precise timing and release of cells to MMP after ischemia requires further study, MMPs, including MMP-9 (gelatinase B), MMP-2 (gelatinase A), and MMP-3 (stromelysin-1), are thought to be key molecules involved in BBB opening and ICB after ischemic stroke (Rosenberg et al., 1995; Castellanos et al., 2003; Wang et al., 2003; Suzuki et al., 2007; Mishiro et al., 2012; Jickling et al., 2014) together with other brain proteases (i.e., plasmin, endogenous t-PA, and urokinase; Wang and Shuaib, 2007). Claudin-5 and occludin, components of the TJs, contain extracellular MMP cleavage sites and are a direct substrate of MMPs (Wachtel et al., 1999; Yang et al., 2007), suggesting that MMPs can degrade TJs directly. As the structural disruption of the interaction between occludins and actin filaments can lead to the perturbation of paracellular permeability (Madara et al., 1986), the degradation of occludin by MMPs is likely to trigger BBB opening. MMPs also degrade components of basement membrane and contribute to BBB impairment, vasogenic edema and hemorrhagic transformation (Rosell et al., 2008). MMP-9 especially is believed to play a major role in BBB disruption during ischemic stroke because ischemic stress induces MMP-9 and the plasma MMP-9 concentration, which strongly correlate with patients’ stroke severity (Horstmann et al., 2003; Jin et al., 2010; Reuter et al., 2013).

MMP-3 is also thought to be involved in ICB due to delayed rt-PA treatment. MMP-3 is produced by pericytes (Yang et al., 2013) and ECs (Suzuki et al., 2007) after ischemic stroke. The increase in ICB caused by the delayed rt-PA treatment was impaired in mice with a gene deficiency of MMP-3, and a broad spectrum MMP-inhibitor suppressed ICB in wild-type mice but not in MMP-3 deficient mice (Suzuki et al., 2007). MMP-3 can be activated by plasmin (Lijnen, 2001), and it has a broad-spectrum substrate specificity, including pro-MMP-9, which is activated by limited cleavage (Nagase and Woessner, 1999). Furthermore, rt-PA treatment induced MMP-3 selectively in ECs at the ischemic damaged area in a mouse stroke model (Suzuki et al., 2007), and MMP-3 was increased in a postmortem human stroke brain (Jickling et al., 2014). These findings indicate that MMP-3 is also involved in degrading the barrier of blood vessels and contributing to ICB.

### Increase in BBB Permeability by Paracellular Transport and Transcytosis

rt-PA treatment is thought to enhance ICB via the acceleration of BBB disruption after ischemic stroke. However, there is still a possibility that rt-PA treatment enhances BBB permeability without BBB disruption after ischemic stroke.

The BBB is composed of blood vessels whose ECs display extremely low rates of transcellular vesicular transport (transcytosis) due to pinocytic activity (Reese and Karnovsky, 1967; Pun et al., 2009; Saunders et al., 2012; Siegenthaler et al., 2013). In concert with pericytes and astrocytes, this unique brain endothelial barrier seals the central nervous system (CNS) and controls substance influx and efflux (Armulik et al., 2010; Bell et al., 2010; Daneman et al., 2010). BBB permeability is regulated in response to various stimulators or stressors, which can exert beneficial or deleterious effects on the brain depending on the context, timing, and functional cellular outcomes of signaling (Roux and Couraud, 2005). BBB permeability can be increased via two processes. The first is paracellular transport, which is associated with loosening the TJs between ECs. The expression of occludin, a component of TJs, is highly suppressed by a number of pathological stresses, including oxidative stress, and a decrease in the occludin expression results in the increase in BBB permeability (Ramirez et al., 2009; Lochhead et al., 2010). To enhance paracellular transport, rt-PA seems to decrease occludin through ROS generation associated with reperfusion and/or through the activation of MMPs.

The second process is transcytosis, which is consistent with endocytosis and involves vesicle transport to the opposite side of the cell and exocytosis. Peripheral ECs display active vesicle trafficking to deliver nutrients to peripheral tissues, whereas CNS ECs express transporters to selectively traffic nutrients across the BBB (Saunders et al., 2012; Siegenthaler et al., 2013). However, it is still unclear when and how ECs are transported by transcytosis.

### VEGF

**Figure 1:**
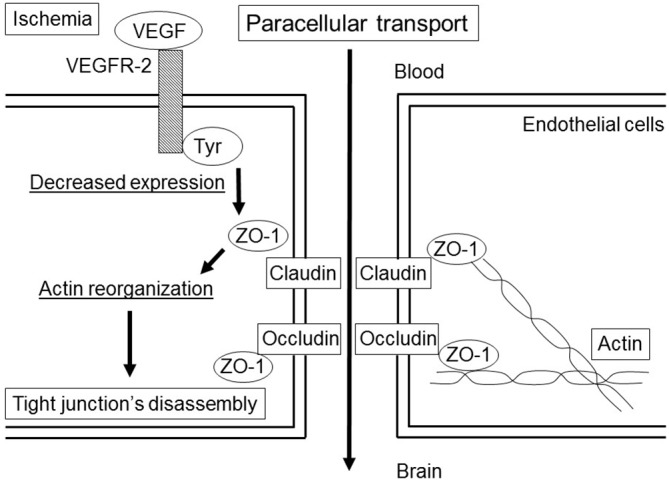
**Paracellular transport in the blood-brain barrier (BBB) by the vascular endothelial growth factor (VEGF) under ischemic condition.** The binding of VEGF to the VEGF receptor-2 (VEGFR-2) induces the activation of tyrosine kinase (Tyr), which leads to a decrease in the expression of zonula occludens (ZO)-1. The changes in ZO-1 correlate with small changes in action distribution, which induce disassembly of the tight junction (TJ).

VEGF stimulates endocytosis and transcytosis (Horowitz and Seerapu, 2012; Nakayama and Berger, 2013). VEGF binds to two receptor-coupled protein tyrosine kinases (Tyr), VEGF receptor 1 (VEGFR-1, Flt-1) and VEGFR-2 (i.e., fetal liver kinase 1 [Flk-1] or the kinase insert domain receptor). VEGF regulates the dissociation of TJs under ischemic condition (Fischer et al., 2002; Figure 1) and endocytosis (Horowitz and Seerapu, 2012), and the subsequent increase in vessel permeability through VEGFR-2 activation, which contributes to cerebral swelling at the early stage after ischemic stroke (Abumiya et al., 2005). VEGF also has a fundamental role in vascular remodeling and angiogenesis by increasing EC proliferation, migration, and microvascular hyperpermeability (Brown et al., 1992). The downstream elements of VEGFR-2 signaling include the Ras/Raf/MEK pathway, which leads to EC proliferation; PI3K-AKT/PKB pathway, which supports EC survival; and p38/MAPK-HSP27 pathway, which promotes EC migration (Obermeier et al., 2013). Vascular remodeling is an important component of recovery after stroke, although it makes vessels to be leakier and prone to intracranial hemorrhage (Durukan et al., 2009). Thus, they may also promote intracranial hemorrhage.

VEGF is expressed in the normal adult brain, mainly in epithelial cells of the choroid plexus, as well as in astrocytes and neurons, such as granule cells of the cerebellum (Monacci et al., 1993; Marti and Risau, 1998). VEGF expression is regulated by extensive signaling pathways. Among them, hypoxia is a strong inducer of VEGF messenger (m)-RNA expression in many cells *in vitro* and *in vivo* (Banai et al., 1994; Ikeda et al., 1995; Kovács et al., 1996; Hayashi et al., 1997). The two transcription factors, hypoxia-inducible factor-1 (HIF-1) and HIF-2 are involved in the regulation of VEGF expression. HIF-1 is composed of a hypoxia-regulated α-subunit and a β-subunit, and is a basic helix-loop-helix heterodimeric transcription factor activated by reduced oxygen tension (Wenger and Gassmann, 1997). HIF-1α is continuously produced and rapidly degraded under normoxia (Sharp and Bernaudin, 2004), whereas it is degraded slowly under hypoxia, and allowed rapid accumulation and binding to hypoxia-responsive elements (Shi, 2009). HIF-2, a homolog of HIF-1, is also involved in the regulation of VEGF gene expression (Ema et al., 1997). HIF-2 has an additional role in the regulation of VEGFR-2 (Kappel et al., 1999). Although VEGF is induced in ECs through HIF-1α (Tang et al., 2004), and the expression of HIF-1 and HIF-2 are increased in the border area of ischemic stroke (Marti et al., 2000), the mechanisms by which VEGF gene expression is regulated during cerebral ischemia remain unclear.

One mechanism for the increase in VEGF secretion by cells exposed to ischemia is an increase in its transcription rate mediated by the binding of HIF-1 to a hypoxia-responsive element in the 5′-flanking region of the VEGF gene, as is observed in PC12 cells (Levy et al., 1995), bovine pulmonary artery ECs (Liu et al., 1995), and Hep3B cells (Forsythe et al., 1996). Other mechanisms include the increase in VEGF mRNA stability (Ikeda et al., 1995) and efficient translation of VEGF mRNA through an internal ribosome entry site (Stein et al., 1998).

### rt-PA, HIF-1, and VEGF

rt-PA increases VEGF expression in bone marrow-derived myeloid cells, cultured cerebral cortical neurons, and ECs (Ohki et al., 2010; Wu et al., 2012; Duan and Ni, 2014; Suzuki et al., 2015). In addition, rt-PA affects HIF-1α regulation. rt-PA induces HIF-1α accumulation in the ischemic brain and accelerates HIF-1α accumulation mediated by the mammalian target of rapamycin in cultured neurons (Wu et al., 2012). rt-PA treatment after ischemia does not enhance the expression of VEGF through the nuclear accumulation of HIF-1α in a transformed mouse brain EC line, bEnd.3 (Suzuki et al., 2015); instead, the overexpression of t-PA stimulates VEGF expression in ECV304, a human immortalized EC due to the stimulation of ERK/p38 signaling pathways (Duan and Ni, 2014). These findings suggest a possibility that rt-PA accelerates the expression of VEGF via HIF-1α or the signal pathway for the upregulation of HIF-1α in parallel without VEGF induction.

The inhibition of VEGF signaling reduces rt-PA-related ICB (Kanazawa et al., 2011; Suzuki et al., 2015), suggesting that VEGF has a deleterious role in ICB. Similarly, VEGF administered within 1–24 h from stroke onset increases in the rate of BBB breakdown and hemorrhagic transformation, and the size of infarction in rodents (Zhang et al., 2000; Abumiya et al., 2005). Taken together that VEGF increases microvascular permeability to blood plasma proteins within minutes after its administration (Dvorak et al., 1995), the induction of VEGF after ischemic stroke may enhance the detrimental effects. In contrast, VEGF administered 48 h from stroke onset enhances angiogenesis and improves neurologic recovery, and improves cerebral blood flow 28 days after stroke (Zhang et al., 2000). The effect of VEGF on angiogenesis is longer than on permeability. Newly formed vessels in ischemic mouse brains are first visible within 4 days (Dellian et al., 1996) and there is an increase in newly formed vessels in ischemic mouse brains 10 days after VEGF treatment but not in contralateral non-ischemic brains (Zechariah et al., 2013). Thus, VEGF seems to have biphasic roles in stroke, it promotes BBB breakdown and hemorrhagic transformation in the early stage, within 24 h, but promotes BBB integrity and vascular function in the late stage, over 48 h, after ischemic stroke.

### Paracellular Permeability by rt-PA

Increased paracellular permeability is correlated with the disruption of TJs (Kevil et al., 2000; Mark and Davis, 2002; Lee et al., 2004). Until now, however, the role of TJs in vascular permeability of either plasma components or circulating cells was supported by the results of only a few studies (Martìn-Padura et al., 1998; Pedram et al., 2002). During ischemic stroke, temporal hypoxemia for 10 min increased BBB permeability associated with alterations in TJ protein expression (Witt et al., 2003). Accordingly, immunoreactivity of ZO-2 or claudin-5 was significantly reduced in infarct regions compared with non-infarct regions 24 h after ischemia (Fischer et al., 2007). However, according to ultrastructural analyses at 5 and 25 h after ischemia, fluorescein isothiocyanate (FITC)-albumin was extravased around vessels with intact TJs, whereas the endothelium exhibited an enhanced transcellular vesicle trafficking (Krueger et al., 2013). Additionally, the morphology of TJ components identified by antibodies against occludin and claudin-5 appears to be regularly maintained in regions where FITC-albumin massively leaked into the neuropil 25 h after ischemia (Krueger et al., 2013). A conclusive time frame for TJ reassembly following the disruption of ischemia is currently lacking. In Madin-Darby canine kidney cells, permeability is returned to the same levels of the initial condition 5 h after ATP repletion. After ATP depletion for 1 h and repletion for 3 h, occludin was once again found almost exclusively at the level of the TJs. This reversible shift is inhibited by the chelation of intracellular calcium. In contrast, ZO-1 is not significantly altered during ATP depletion or repletion (Ye et al., 1999). However, VEGF specifically down-regulates claudin-5, occludin protein, and mRNA. In the mouse cerebral cortex, the microinjection of VEGF disrupted claudin-5 and occludin, and induced loss of barrier function (Argaw et al., 2009). The continuity of the ZO-1 expression was significantly disrupted during 1.5 h of hypoxia in primary cultures isolated from porcine brain ECs. Furthermore, VEGF alone or α-lipoic acid alone did not change ZO-1 localization in the primary culture, however, VEGF in combination with α-lipoic acid decreased the ZO-1 expression to nearly the same extent as 3 h of hypoxia (Fischer et al., 2002). These results suggest that ischemia increases the paracellular flux via the release of VEGF, which in turn leads to the dislocalization, decreased expression, and enhanced phosphorylation of TJs. As TJ proteins are responsible for the paracellular permeability across the BBB, VEGF induced by ischemia may increase paracellular permeability of BBB via suppression of the expression of TJ proteins in the early stage of ischemic stroke (Fischer et al., 2002; Argaw et al., 2009, 2012). However, it is possible that BBB permeability is increased by TJs loosening in the early stage of ischemic stroke because a study found intact TJs at 24 h after stroke (Krueger et al., 2013).

Delayed rt-PA treatment enhances the fragmentation of occludin and claudin-5 24 h after middle cerebral artery (MCA) occlusion in rats (Won et al., 2014), and reduces claudin-5 at 24 h (Ishiguro et al., 2010) or occludin and ZO-1 at 48 h after ischemia in mice (Mishiro et al., 2012). Furthermore, it was found that blood levels of TJ proteins were higher in patients with hemorrhagic transformation than in those without hemorrhagic transformation (Kazmierski et al., 2012). These findings indicate that delayed rt-PA treatment results in the enhancement of ICB due to stroke by the loss of TJ proteins at a relatively later stage, over 24 h, on ischemic stroke. However, the role of TJ proteins in early ICB is still unclear. Delayed rt-PA treatment may enhance the BBB permeability through the paracellular pathway by the degradation of TJ proteins without BBB breakdown as well as ischemia without rt-PA treatment.

### rt-PA Treatment and Endocytosis

**Figure 2:**
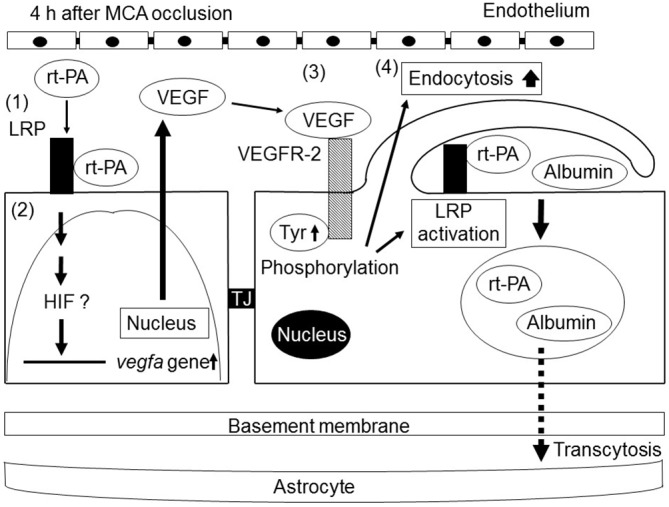
**Schematic of the mechanisms of the increase in BBB permeability by recombinant tissue-type plasminogen activator (rt-PA) treatment after ischemic stroke.** rt-PA activates the low-density lipoprotein receptor-related protein (LRP), which is upregulated in endothelial cells (ECs) by ischemic stress (1). The activation of LRP induces the transcriptional upregulation of VEGF. Secreted VEGF binds to VEGFR-2 on the surface of ECs through an autocrine mechanism and induces its phosphorylation (3). The activation of VEGFR-2 leads to an increase in endocytosis and to the activation of LRP, resulting in enhanced BBB permeability by endocytosis and subsequent transcellular transport of proteins into cerebroparenchyma (4). Tyr, TJ.

The intravenous treatment of rt-PA does not increase either albumin extravasation or ICB in naive mice (Cheng et al., 2006; Su et al., 2008; Suzuki et al., 2015). Additionally, the intraventricular injection of rt-PA does not increase Evans blue extravasation in sham-operated mice (Yepes et al., 2003). In contrast, an intravenous injection of rt-PA slightly increases the Evans blue extravasation in native mice (Turner and Vink, 2012). Furthermore, an intravenous injection of biotinylated rt-PA with fluorescent dextran (77 kDa) is detected the extravasation of rt-PA in the brain parenchyma of nonlesioned animals (Benchenane et al., 2005), suggesting that rt-PA itself and other plasma molecules can cross the intact BBB via transcytosis. Similarly, we observed that delayed rt-PA treatment dramatically increased endocytosis of cerebral ECs at the ischemic border region together with an increase in the existence of gold-labeled bovine serum albumin (BSA) administered intravenously at the vascular lumen, inside ECs, in the basement layer of the ECM and extravascular space without obvious TJ defects in mice (Suzuki et al., 2015). This indicates that delayed rt-PA treatment increased extravasation of BSA at the ischemic border region in the early period after MCA occlusion by the additional acceleration of transcytosis rather than by the degradation of vascular structures. It is likely that the administration of rt-PA accelerates extravasation of phagocytic vesicles, including rt-PA, and interacting plasma plasminogen in the parenchyma by the upregulation of transcytosis increases the likelihood of a plasmin-dependent BBB alteration at the perivascular space. Because this study does not provide direct evidence for the involvement of exocytosis, another component of the transcytosis process, in the ischemic border region of the extravasation of BSA, there is still the possibility that endothelial endocytosis is independent of BBB opening. Additionally, transcytosis may possibly be involved in the increase in BBB permeability by the delayed treatment of rt-PA after ischemic stroke (Figure 2).

### Endocytosis and the Multifunction of LRP

As previously described, LRP is a scavenger receptor that binds a variety of biological ligands associated with the ECM, and it is a major binding protein of rt-PA (Bu et al., 1992). LRP acts as a membrane receptor of rt-PA, and its activation induces the expressions of MMPs and VEGF (Wang et al., 2003; Suzuki et al., 2009, 2015). The invasion of carcinoma cells is also decreased by LRP silencing with RNA interference despite a strong stimulation of pericellular MMP-2 and urokinase-type plasminogen activator proteolytic activities (Dedieu et al., 2008). However, the precise role of LRP in the regulation of ECM remodeling is still unclear.

During endocytosis, LRP does not act alone, as it has membrane partners that vary according to numerous parameters, including the cell origin, ECM composition, and pathological conditions (Etique et al., 2013). LRP-mediated endocytosis of soluble ligands is usually followed by intracellular lysosomal routing and catabolism. LRP emerges as an endocytic receptor regulating cellular matrix attachment sites and coordinating the balance of adhesion/deadhesion. It has been known that a small number of transmembrane proteins are associated with LRP. As this association is thought to be involved in the endocytosis and subsequent turnover of the membrane proteins, the mechanisms are insufficient to be understood. As LRP is a major binding protein of t-PA, the administration of rt-PA may stimulate ECs and accelerate the endocytosis of plasma proteins via LRP and subsequent extravasation of proteins into the parenchyma.

### rt-PA, Plasmin, and the Substrate for Plasmin

It is unclear whether the involvements of rt-PAs during ischemia, except for clot lysis, are associated with plasmin. Plasminogen is essentially present in both blood and the brain under most pathologic brain scenarios, especially together with rt-PA during its utilization in ischemic stroke. Plasminogen is exclusively localized in neurons of the cerebral cortex, hippocampus, hypothalamus, and the cerebellum in rodents (Tsirka et al., 1997; Basham and Seeds, 2001; Taniguchi et al., 2011). Hence, it is not likely to be obviously assumed that brain-derived plasminogen is activated at the BBB during stroke by endogenous t-PA. Blood-derived plasminogen may be more readily available by passing the BBB, and it may be activated at the BBB under an ischemic condition. Therefore, brain-derived plasminogen in the neuronal pathology may not be completely associated with BBB permeability.

As previously described, plasmin can directly degrade basement membrane components or cause degradation of basement membrane components via the activation of MMPs (Lijnen, 2001), which possess similar basement membrane dismantling capabilities and damages the TJs (Jin et al., 2010). rt-PA treatment did not alter ICB associated with stroke in mice deficient in plasminogen and MMP-3, suggesting that plasmin may be required to activate MMP-3 by rt-PA in ECs during stroke (Suzuki et al., 2007).

Plasmin (and plasminogen) binds a wide array of cell-surface receptors or binding proteins and cleaves a variety of biologic substrates (Kwon et al., 2005; Miles and Parmer, 2013). As a result of this capacity, plasmin has been understood to play a role in many cellular responses, including cell migration, wound healing, tissue remodeling, apoptosis, cancer invasion, cancer metastasis, and inflammation and immunity, as extensively reviewed elsewhere (Kwon et al., 2005; Syrovets et al., 2012; Miles and Parmer, 2013). Together, these characters attribute a position to plasmin as a sound candidate that participates in the remodeling of cerebral blood vessels, especially during stroke, when the BBB weakens and blood components gain access to the BBB. The direct intracortical injection of concentrated plasmin resulted in substantial lesion formation 6 h later, accompanied by the oxidation of proteins and DNA, degradation of occludin and collagen IV (basement membrane), and elevation of MMP-9, without the loss of ECs (Lukic-Panin et al., 2010). These findings indicate that plasmin can evidently influence TJ proteins and basement membrane. Therefore, it is likely that the administration of rt-PA accelerates the likelihood of a plasmin-dependent BBB alteration.

## Conclusions

Delayed rt-PA treatment increases BBB permeability through a number of mechanisms. Although the mechanisms depend on the degree of cell damage after ischemia, rt-PA has the possibility of increasing BBB permeability without compromising BBB integrity and causing subsequent BBB breakdown. rt-PA increases BBB permeability via the induction of VEGF, which at least partially mediates the subsequent increase in endothelial endocytosis. Furthermore, an increase in BBB permeability by endocytosis is likely the first step in BBB breakdown by delayed rt-PA treatment combined with ischemic stroke because various plasma proteins in the bloodstream are taken up by the parenchyma across the compromised BBB.

## Author Contributions

YS and NN: planning and writing the review. KU: supervisor.

## Conflict of Interest Statement

The authors declare that the research was conducted in the absence of any commercial or financial relationships that could be construed as a potential conflict of interest.

## Abbreviations

BBB: blood-brain barrier
BSA: bovine serum albumin
CNS: central nervous system
EC: endothelial cell
ECM: extracellular matrix
FITC: fluorescein isothiocyanate
HIF: hypoxia-inducible factor
ICB: intracranial bleeding
LRP: low-density lipoprotein receptor-related protein
MCA: middle cerebral artery
MMP: metalloproteinase
ROS: reactive radical oxide species
rt-PA: recombinant tissue-type plasminogen activator
VEGF: vascular endothelial growth factor
TJ: tight junction
ZO: zonula occludens.
